# Supplementary material for: Advanced Respiratory Models for Hazard Assessment of Nanomaterials—Performance of Mono-, Co- and Tricultures
Source: Nanomaterials (Basel). 2022 Jul 29;12(15):2609. doi: 10.3390/nano12152609 (PMC9370172; doi:10.3390/nano12152609)
Supplement: Supplementary file 1 [file nanomaterials-12-02609-s001.zip › nanomaterials-1817099-supplementary.pdf]

Supplementary Materials

Advanced Respiratory Models for Hazard Assessment of Nanomaterials—Performance of Mono-, Co- and Tricultures

Laura Maria Azzurra Camassa [1,†], Elisabeth Elje [2,3,†], Espen Mariussen [2,4‡], Eleonora Marta Longhin [2], Maria Dusinska [2], Shan Zienolddiny-Narui [1,*] and Elise Rundén-Pran [2,*]

[1] National Institute of Occupational Health in Norway, 0033 Oslo, Norway; laura.camassa@stami.no
[2] NILU—Norwegian Institute for Air Research, 2027 Kjeller, Norway; eel@nilu.no (E.E.); espen.mariussen@fhi.no (E.M.); eml@nilu.no (E.M.L.); mdu@nilu.no (M.D.)
[3] Institute of Basic Medical Sciences, Department of Molecular Medicine, University of Oslo, 0372 Oslo, Norway
[4] Norwegian Institute of Public Health, FHI, 0456 Oslo, Norway
* Correspondence: shan.narui@stami.no (S.Z.-N.); erp@nilu.no (E.R.-P.); Tel.: +47-2319-5284 (S.Z.-N.); Tel.: +47-6389-8237 (E.R.-P.)
† These authors contributed equally to this work.
‡ Present address.

S1.1. Cell cultures

The formulation of the different types of culture medium is summarized in Table S1. The passage numbers used for cells in different culture models are summarized in Table S2, and the density of the cell types in Table S3.

Table S1. Cell types and cell media with supplements used for mono-, co- and tricultures. DMEM: Dulbecco's Modified Eagle's Medium. FBS: Fetal bovine serum, hiFBS: heat inactivated FBS (56°C, 30 min), pen/strep: penicillin-streptomycin. RPMI: Roswell Park Memorial Institute Medium. 1% pen-strep equals 100 U/mL penicillin and 100 µg/mL streptomycin. % given as v/v.

Cell Type	Cell Media<br>Lab 1	Cell Media<br>Lab 2
	Monoculture	
A549 epithelial<br>(ATCC® CCL-185™)	DMEM (Sigma, D6046)<br>+ 9% FBS + 1% pen/strep	DMEM low glucose (Gibco 31885023)<br>+ 10% hiFBS + 1% pen/strep
EA.hy926 endothelial<br>(ATCC® CRL2922™)	DMEM high glucose with pyruvate and L glutamine (Gibco 11995-065)<br>+ 9% FBS + 1% pen/strep	DMEM high glucose (Gibco 11965-092)<br>+ 10% hiFBS + 1% pen/strep
THP-1 monocytes<br>(ATCC® TIB-202™)	RPMI1640 (Gibco, A1049101-01)<br>+ 9% hiFBS + 1% pen/strep	RPMI1640 (Gibco, A1049101-01)<br>+ 10% hiFBS + 1% pen/strep
	Coculture/Triculture	
Coculture:<br>A549/EA.hy926<br>and<br>*Triculture:*<br>A549/EA.hy926/dTHP-1	*Apical side:*<br>DMEM low glucose<br>+ 9% FBS + 1% pen/strep<br><br>*Basolateral side:*<br>DMEM high glucose<br>+ 9% FBS + 1% pen/strep<br><br>*ALI conditions basolateral side:*<br>72 % DMEM high glucose + 18 % DMEM low glucose + 9% FBS + 1% pen/strep	*Apical side:*<br>DMEM low glucose<br>+ 10% hiFBS + 1% pen/strep<br><br>*Basolateral side:*<br>DMEM high glucose<br>+ 10% hiFBS + 1% pen/strep<br><br>*ALI conditions basolateral side:*<br>72 % DMEM high glucose + 18 % DMEM low glucose + 10% hiFBS + 1% pen/strep

Table S2. Passage numbers of cells applied in the experiments.

		Monoculture	Coculture	Triculture
**A549**	Lab 1	4, 5, 6, 9, 13, 15	2, 5, 9, 11, 12, 13, 15	13, 16, 19, 21
	Lab 2	5, 7, 9, 11	13, 15, 17	19, 21, 23, 25
**EA.hy926**	Lab 1	-	3, 5, 7, 8, 9, 10, 12	3, 14, 16, 19
	Lab 2	-	5, 7, 9, 11	13, 15, 17, 19
**THP-1**	Lab 1	-	-	6, 9, 12, 15
	Lab 2	-	-	5, 8, 11, 14

Table S3. Cell seeding densities in advanced lung models on PET 1 μm transwell inserts (BD Biosciences, Millipore, Falcon). Constant cell densities were used within each experiment. PET: polyethylene terephthalate.

**Cell type**	**Seeding density (cells/cm²)**		
	Monoculture	Coculture	Triculture
A549	1.1 x 10^5	1.1 x 10^5	1.1 x 10^5
EA.hy926	-	1.1 x 10^5	1.1 x 10^5
dTHP-1	-	-	1.1-2.2 x 10^5

Table S4. Antibodies and staining for confocal microscopy.

		**Supplier**	**Dilution**
**Primary antibodies**	**Host species**		
Anti- Prosurfactant Protein C antibody	rabbit	ab90716 Abcam	1:250
Anti- Zonula Occludens 1 (ZO-1)	rabbit	61-7300 Life technologies	1:250
Anti- Cd11b	mouse	AM32402PU-N-Origene	1:200
**Secondary antibodies**			
Donkey anti-Rabbit IgG (H+L) Highly Cross-Adsorbed Secondary Antibody, Alexa Fluor 488		Life technologies	1:1000
Donkey anti-Mouse IgG (H+L) highly cross-adsorbed secondary antibody, Alexa Fluor 596		Life technologies	1:1000
**Nuclei staining**			
DAPI		Sigma	1:1000

S1.2. NM-300K endotoxin testing

The HEK endotoxin assay can detect as low as 0.01 EU/mL of endotoxin. A 1 unit/mL of endotoxin (EU/mL) is equal approximately 0.1 ng endotoxin/mL of solution. When HEK293 transfected with hTLR2 and hTLR4 were exposed to NM-300K at a concentration of 1 and 10 μg/mL (Fig. S1), they released less than 1 unit/mL of endotoxin (EU/mL). This value is comparable with the HEK293 hTLRnull used as a negative control. On the contrary, HEK293 hTLR2 and hTLR4, showed an extremely high concentration of endotoxin released (positive control) when exposed to their respective agonists LTA and LPS (Fig. S1, left). Higher dosage of NM-300K is not vital for HEK 293 cells (data not shown). HEK293 TLRnull, HEK293 hTLR2 and hTLR4 viability was measured by AB assay for validation of the assay (Fig. S1, right). HEK293 cell viability was affected by transfection of the hTLR2 and hTLR4 but this was independent of the chosen concentrations of NM-300K. Relative viability (%) was measured by AlamarBlue. The fluorescence intensity is proportional to the number of living cells. Cells were incubated with a volume of 200 μL/well of respective medium containing 10 % v/v AB solution (Sigma-Aldrich) at 37°C and 5% CO_2 for 1-1.5 h. Four replica wells were included per condition. Aliquots of 100 μL were taken from each well and transferred into a new 96 well plate to measure the fluorescence intensity (ex.530 nm, em.590 nm). HEK293, HEK293 hTLR2 and hTLR4 cells

viability was not influenced by 1 and 10 μg/mL NM-300K. A decrease in cell viability appears to be dependent by the different cell medium composition more than the exposure to NMs.

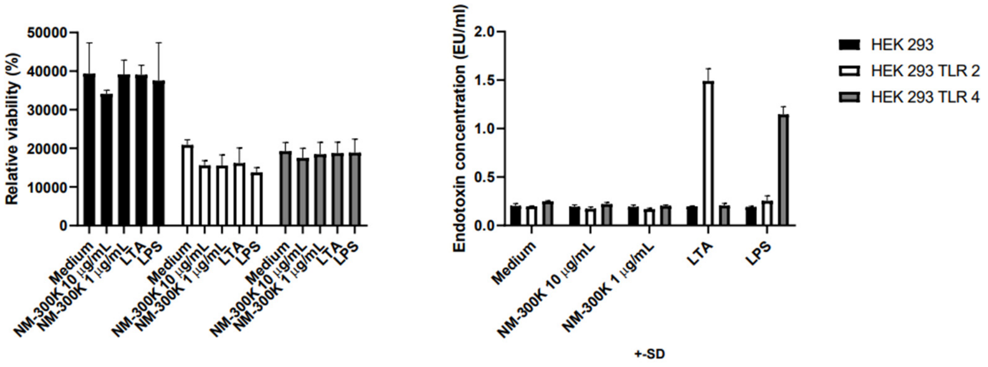

Figure S1. HEK293 AlamarBlue (AB) and endotoxin test 24 hours postexposure with NM-300K. (Left) AB assay: HEK293, HEK293 hTLR2 and hTLR4 cell viability was not influenced by 1 and 10 μg/mL NM-300K. (Right) Exposure with NM-300K did not show any relevant endotoxin concentration (EU/mL) at a concentration of 1 and 10 μg/mL. Endotoxin level followed by exposure showed a level comparable to the negative control cells (hTLRnull). Note the response of HEK293 hTLR2 and hTLR4 when exposed to their respective agonist LTA and LPS. Results are presented as mean with standard deviation of 4 replica samples.

NM-300K nanoparticles at the concentration of 0.5 and 50 μg/mL was also examined for endotoxins with LAL kinetic QCL endotoxin chromogenic test (Lonza). LAL assay is extremely sensitive and detect as little as 0.1 EU/mL (approx. 0.01 ng endotoxin per mL). No considerable endotoxin concentration was detected in any of the nanoparticles and dispersing medium analysed.

NM-300K has a yellow-brown colour with absorbance peak at about 410 nm (Elje et al 2020) and is likely to interfere with the LAL assay when tested at high concentrations, as the assay includes absorbance reading at 405 nm. Our results are at low concentrations, thus, endotoxin contamination cannot be completely excluded, as the sample could be too diluted and below the sensitivity of the test.

S1.3. Calculation of nominal concentrations for exposure in the VITROCELL® system

The deposition efficiency in the VITROCELL® system was estimated to 50 % in preliminary experiments (results not shown). This was used for calculation of nominal concentrations of NM-300K according to the formulas below.

Stock concentration, undiluted = C_{stock} = 10 mg/mL = 10 000 μg/mL

Stock concentration, diluted = $C_{diluted}$ = 1 mg/mL = 1000 μg/mL

Nebulizing volume of sample = V_{sample} = 300 μL

Total deposition area = A_{total} = 145 cm^2 [39]

Deposition efficiency (DE) = 50 % (preliminary experiments)

Nominal concentration low = $C_{nominal\ low}$

Nominal concentration high = $C_{nominal\ high}$

$$C_{nominal\ low} = \frac{C_{diluted} \times V_{sample}}{A_{total}} \times DE = \frac{1000\ \frac{\mu g}{ml} \times 0{,}3\ ml}{145\ cm^2} \times 0{,}50 = 1{,}03\ \mu g/cm^2$$

$$C_{nominal\ high} = \frac{C_{stock} \times V_{sample}}{A_{total}} \times DE = \frac{10000\ \frac{\mu g}{ml} \times 0{,}3\ ml}{145\ cm^2} \times 0{,}50 = 10{,}34\ \mu g/cm^2$$

S1.4. Characterization of NM-300K

The Ag concentration of NM-300K was measured by ICP-MS at Lab 1, and the size distribution of NM-300K was measured by DLS at both laboratories. A summary of the overall characterization is given in Table S5. DLS results per sample and laboratory are summarized in Tables S6 and S7 showing the average size of stock dispersion and diluted dispersion (in PBS), respectively. The size of NM-300K was similar in stock dispersion and after dilution in PBS. For stock dispersions prepared at Lab 1, one dispersion had a smaller size compared to the others. No relationship was found between mean diameter and delivered energy during sonication (results not shown) and is not believed to be the explanation for differences in Z-ave.

Table S5. Characterization of NM-300K dispersions. Measurements of NM-300K stock dispersion with nominal concentration 10 mg/mL performed at Lab 1 and Lab 2. Ag content in stock was measured by ICP-MS. The dispersion was diluted 1:100 in pure water for size (by intensity) and ZP analysis. Presented are mean values with SD from three independent experiments (n=3), except for Ag concentration where n=7 and n=2, and Z-ave (Lab 1) where n=9. DLS: dynamic light scattering, Z-ave: average hydrodynamic diameter, PDI: polydispersity index, ZP: zeta potential, ICP-MS: inductively coupled plasma mass spectrometry.

		Lab 1	Lab 2
Ag content (mg/mL)	**Ag concentration**	7.20 ± 0.87 (n=7)	-
	**Ag < 3 kDa fraction**	0.29 ± 0.01 (n=2)<br>3.6 % ± 0.1 %	-
DLS	**Z-ave (nm)**	130.7 ± 23.2	57.5 ± 5.7
	**PDI**	0.380 ± 0.037	0.343 ± 0.067
	**ZP (mV)**	-17.1 ± 2.8	-

Tab S6: Size distribution of NM-300K diluted 1:100 in ultrapure H_2O, measured by DLS (by intensity) in two laboratories. A Zetasizer Ultra Red with measurement angle 174.4° was used at Lab 1, and a Zetasizer Nano ZS with measurement angle 173° was used at Lab 2. Results are average of 3-5 steps per sample. The stock ID corresponds to Supplementary table 5.

**Laboratory**	**Stock ID**	**Z-Ave**	**PdI**	**Main peak**	**Smaller peak**	**Larger peak**	**Main peak**	**Smaller peak**	**Larger peak**
		nm	a.u.	nm	nm	nm	%	%	%
Lab 1	Stock1	75.6	0.442	108.4	5.1	4353.0	92.9	1.8	5.3
Lab 1	Stock2	146.7	0.413	216.8		4570.0	96.9		3.1
Lab 1	Stock3	137.0	0.387	227.0	6.5		99.6	0.4	
Lab 1	Stock4	136.9	0.402	222.4	18.6	4591.0	95.1	2.6	2.2
Lab 1	Stock5	128.2	0.388	203.1	10.5		98.6	1.4	
Lab 1	Stock6	115.5	0.354	176.4	18.2		96.4	3.6	
Lab 1	Stock7	146.0	0.370	234.3			100.0		
Lab 1	Stock8	143.2	0.321	211.1		1596.3	99.1		0.9
Lab 1	Stock9	147.6	0.345	215.0		3247.2	98.1		1.9
	Average	130.7	0.380	201.6	11.8	3671.5	97.4	2.0	2.7
	SD	23.2	0.037	38.7	6.4	1284.9	2.3	1.2	1.7
Lab 2	Stock1	54.4	0.299	81.4	8.0		94.9	5.1	
Lab 2	Stock2	54.0	0.310	80.9	7.8	4566.0	94.4	5.4	0.2
Lab 2	Stock3	64.1	0.420	103.8	10.6	4288.0	93.1	5.3	1.6
	Average	57.5	0.343	88.7	8.8	4427.0	94.1	5.3	0.9
	SD	5.7	0.067	13.1	1.6	196.6	0.9	0.2	1.0

Table S7 Size distribution of NM-300K diluted 1:100-1:10 in PBS, measured by DLS (by intensity) in two laboratories. A Zetasizer Ultra Red with measurement angle 174.4° was used at Lab 1, and a Zetasizer Nano ZS with measurement angle 173° was used at Lab 2. Results are average of 3-5 steps per sample. The stock ID corresponds to Table S4.

Laboratory	Stock ID	Z-Ave	PdI	Main peak	Smaller peak	Larger peak	Main peak	Smaller peak	Larger peak
		nm	a.u.	nm	nm	nm	%	%	%
Lab 1	Stock1	76.0	0.434	117.4	1653.2	2451.4	91.3	2.9	5.8
Lab 1	Stock8	144.5	0.359	224.0	0.0	1218.0	97.5	0.0	2.5
Lab 1	Stock9	151.4	0.368	230.3	1231.7	1572.3	96.7	2.5	0.7
Lab 2	Stock1	57.4	0.298	84.7	8.2	4694.0	96.0	3.8	0.2
Lab 2	Stock2	60.9	0.382	92.4	9.6	4285.0	97.2	1.4	1.2
Lab 2	Stock3	69.0	0.427	114.1	8.4	3591.0	93.9	2.2	3.9

S1.5. Stability of NM-300K in physiological buffers

To compare the stability of NM-300K in different buffers to use in the cloud system, the NM-300K was diluted 1:10 in pure water, PBS and HBSS with and without $CaCl_2$ and $MgCl_2$, before dilution 1:10 in pure water and measurement of hydrodynamic diameter by DLS. The NMs were stable in size upon dilution in different buffers (Table S8).

Table S8. Characterization of hydrodynamic diameter by DLS of NM-300K in different buffers (Lab 1). Results are shown as mean with SD of n=3 independent experiments. *n=2. PBS: Phosphate buffered saline. HBSS: Hank's balanced salt solution. -, without $CaCl_2$ and $MgCl_2$. +, with $CaCl_2$ and $MgCl_2$. PDI, polydispersity index.

	Pure water	PBS -	PBS + (*)	HBSS -	HBSS +
Z-ave (nm)	128.5 ± 30.7	123.9 ± 41.8	129.3 ± 33.7	120.6 ± 44.7	122.0 ± 42.9
PDI (nm)	0.359 ± 0.075	0.385 ± 0.037	0.294 ± 0.062	0.435 ± 0.150	0.388 ± 0.057

S1.6. AlamarBlue assay on cultures exposed to different physiological buffer solutions

The relative cell viability of A549 and EA.hy926 cells in cocultures after exposure to aerosolized buffers, was investigated by the AlamarBlue assay. Results are shown in Fig. S2 and explained in the main document.

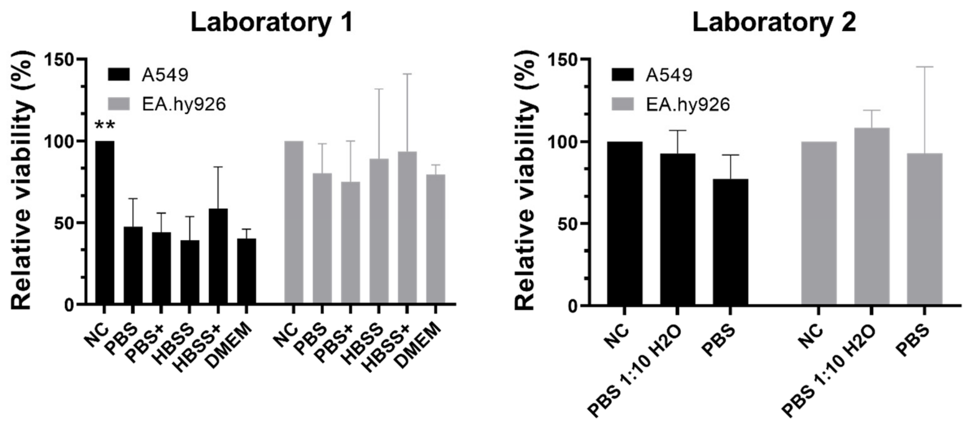

Figure S2. Relative viability measured by the AlamarBlue assay in A549/EA.hy926 cocultures exposed to buffer solutions at the air-liquid interface (ALI), performed at Lab 1 (left image) and Lab 2 (right image). The viability of A549 cells in cocultures was decreased after buffer exposure. Results are shown as mean with standard deviation (SD) of 3 independent experiments, each with duplicate cell culture inserts. Statistically significant differences compared to PBS were analyzed by ordinary one-way ANOVA with multiple comparisons post-test Dunnet´s, and are indicated by * p <0.5, ** p<0.1, and *** p<0.01. NC: negative control (incubator control), PBS: phosphate buffered saline, HBSS: Hanks' balanced saline solution, DMEM: Dulbecco's modified Eagle medium, +: with CaCl2/MgCl2.
